# ZNF703 promotes triple-negative breast cancer cells through cell-cycle signaling and associated with poor prognosis

**DOI:** 10.1186/s12885-022-09286-w

**Published:** 2022-03-02

**Authors:** Xi Zhang, Xin Mu, Ou Huang, Zhitang Wang, Jialin Chen, Debo Chen, Gen Wang

**Affiliations:** 1 Department of Breast Oncology, The First Hospital of Quanzhou Affiliated to Fujian Medical University, Anji Rd, 362000 Quanzhou, China; 2 Comprehensive Breast Health Center, Ruijin Hospital, Shanghai Jiaotong University School of Medicine, 200025 Shanghai, China; 3 Department of Urology, The Second Affiliated Hospital of Fujian Medical University, 362000 Quanzhou, China; 4 Department of Pharmacology, School of Pharmacy, Fujian Provincial Key Laboratory of Natural Medicine Pharmacology, Fujian Medical University, University Town, 1 Xue Yuan Road, 350122 Fuzhou, China

**Keywords:** ZNF703, Triple-negative breast cancer, Cell proliferation, Cell cycle, Prognosis

## Abstract

**Background:**

The oncogenic drivers of triple-negative breast cancer (TNBC), which is characterized by worst prognosis compared with other subtypes, are poorly understood. Although next-generation sequencing technology has facilitated identifying potential targets, few of the findings have been translated into daily clinical practice. The present study is aimed to explore *ZNF703 (Zinc finger 703)* function and its underlying mechanism in TNBC.

**Methods:**

ZNF703 expressions in tissue microarray were retrospectively examined by immunohistochemistry. The cell proliferation by SRB assay and colony formation assay, as well as cell cycle distribution by flow cytometry were assessed. The protein levels associated with possible underlying molecular mechanisms were evaluated by western blotting. Kaplan-Meier analysis was used to plot survival analysis.

**Results:**

Our data suggest that *ZNF703* expressed in 34.2% of triple-negative human breast tumors by immunohistochemistry. *In vitro*, *ZNF703* knockdown had potent inhibitory effects on TNBC cell proliferation and cell cycle, with cyclin D1, CDK4, CDK6, and E2F1 downregulated, while Rb1 upregulated. Moreover, Kaplan-Meier analysis showed that high mRNA expression of *ZNF703* was correlated to worse overall survival (*HR* for high expression was 3.04; *95% CI*, 1.22 to 7.57, *P* = 0.017).

**Conclusions:**

Taken together, the results identified that targeting *ZNF703* contributed to the anti-proliferative effects in TNBC cells, due to induced G1-phase arrest. This study is the first to identify ZNF703 as a potentially important protein that is involved in TNBC progression.

**Supplementary Information:**

The online version contains supplementary material available at 10.1186/s12885-022-09286-w.

## Introduction

Triple-negative breast cancer (TNBC), defined as lack of expression of estrogen receptor α (ERα), progesterone receptor (PR) and human epidermal growth receptor 2 (HER2) / erb-b2 receptor tyrosine kinase 2 (ERBB2), which does not benefit from routine targeted therapies and is associated with poor outcome [1, 2], is the most aggressive subtype of breast cancer. Although patients with early stages of TNBC may be cured with chemotherapy, median overall survival is rather limited in those who suffer from recurrent or metastatic diseases [3, 4]. The inner mechanisms that drive the abnormal proliferation of TNBC are still poorly understood; targeted agents are still to be developed and could result in improved overall survival for TNBC patients [5–7]. Most early TNBC patients are treated with chemotherapy, including anthracyclines, paclitaxel, or platinum. Metastatic TNBC patients are likely to be resistant to chemotherapy and have little choices to be treated with specific targeted therapies to prolong survival [8, 9]. Clinical trials have demonstrated few effective targeted drugs, including PARP inhibitors [10], PD-1 or PD-L1 inhibitors [11–13]. TNBC encompasses molecularly different subgroups [14]; however, molecular-subgroup-based therapies have not been established.

Scientists have explored about *ZNF703 (Zinc finger 703)* in cancer fields. It is a transcriptional factor, which is also an oncogene in luminal B breast cancer, identified by genome-wide measurements of DNA copy number using comparative genomic hybridization [15, 16]. Some studies [17] have used integrated analysis of copy number and gene expression in a discovery and validation set of almost 2000 primary breast tumors, in which copy number changes of *ZNF703* are very obvious and common in breast tumors, secondary to *ERBB2* and *CCND1*. Therefore, *ZNF703* is a new and very important oncogene in breast cancer, and it should be considered as a therapeutic target in ~15% of breast tumors [18]. The rearrangements of individual tumors in a cohort of 560 breast cancers were systematically investigated, and it reveals that simultaneous amplification of chromosome 8—*ZNF703*/*FGFR1*—and chromosome 11—*CCND1*—where there is a chromosome 8–chromosome 11 translocation, is likely to be an early, critical, initiating event in breast cancer [19]. However, it seems that those amplified genes are not always overexpressed [20].

In the present study, for the first time, we discovered that *ZNF703* was also expressed in part of triple-negative breast cancer, whether in the human tumor specimens or cancer cell lines. Here we assessed, for the first time to our knowledge, the activity of *ZNF703* inhibition and the underlying mechanisms in TNBC cell lines: MDA-MB-468 and BT549, as well as analyzed the relationship between overall survival and *ZNF703* expression in TNBC.

## Materials and methods

### Cell culture, reagents and antibodies

**Fig. 1:**
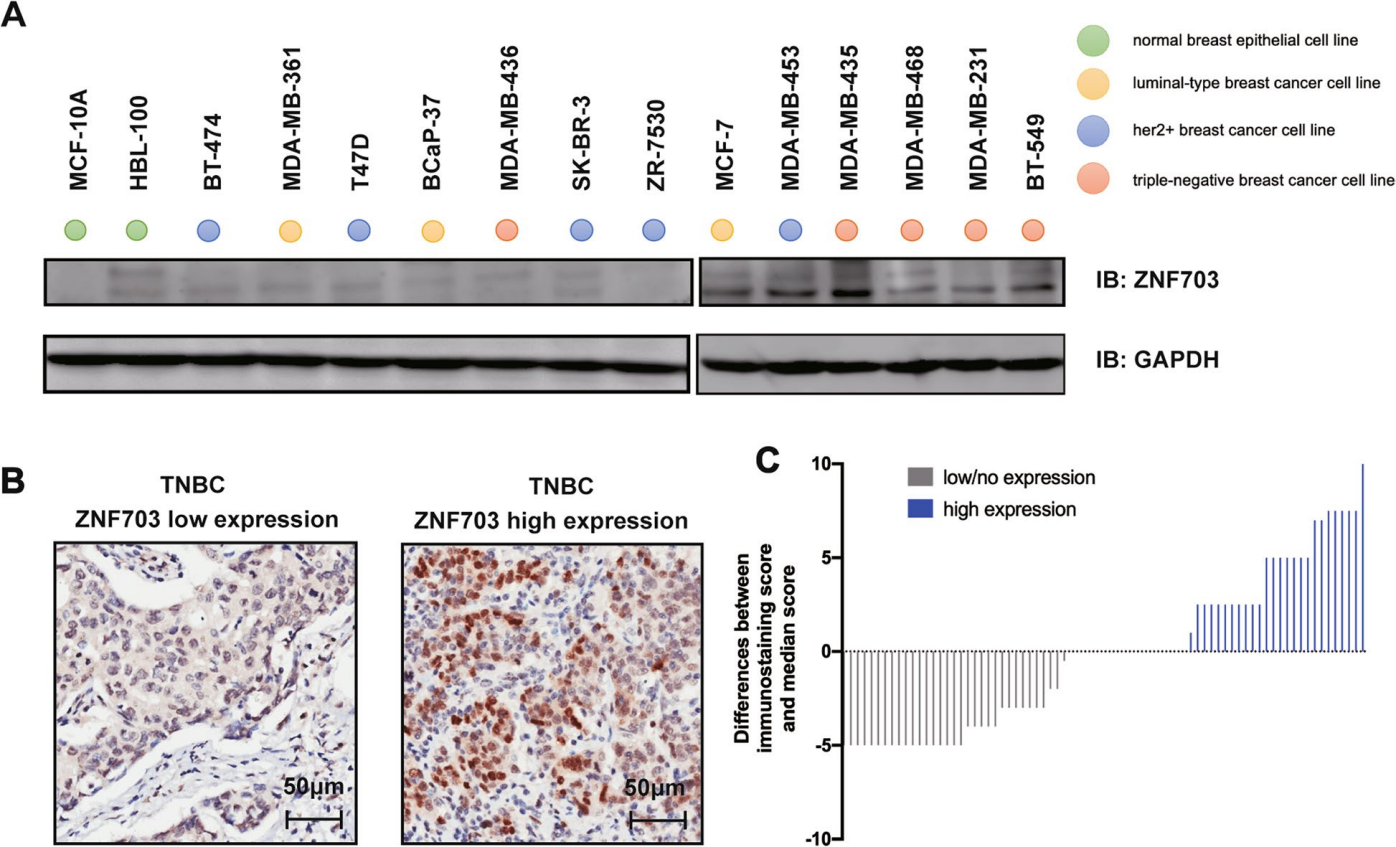
*ZNF703* expression in breast cancer. **A** Immunoblotting (IB) for *ZNF703* in total cell lysates from five triple-negative breast cancer (TNBC) cell lines (red circles), two normal breast epithelial cell lines MCF-10 A, HBL-100 (green circles) and representative examples of other breast cancer subtypes (yellow circles for luminal-type, and blue circles for HER2-positive subtype). A GAPDH antibody was used as a loading control. **B** Representative image of IHC staining for *ZNF703* in TNBC specimens. Left: low *ZNF703* expression; Right: high *ZNF703* expression. The bar represents 50 μm. **C** Immunostaining scores of *ZNF703* in 76 TNBC patients. The vertical axis indicates the differences between the score of each patient and the median score. High expression group was indicated as positive numbers, and low/no expression group was indicated as zero or negative numbers

All breast cancer cell lines were purchased from the American Type Culture Collection (ATCC, Manassas, VA, USA). MDA-MB-468 and BT-549 were cultured in RPMI 1640 medium (Gibco) with 10% Fetal bovine serum (FBS, Gibco) supplemented with 2 mM L-glutamine. Other cell lines were cultured followed by instructions from ATCC guideline. Among them, cell lines were classified into four distinguished subtypes, including normal breast epithelial cell line, luminal-type breast cancer cell line, HER2-positive breast cancer cell line, and triple-negative breast cancer cell line (Fig. 1A).

The antibodies used in this study were as follows: *ZNF703* for Western blot (1:1000 dilution, Abcam, No.ab137054), *ZNF703* for immunohistochemistry (1:50 dilution, Sigma-Aldrich, St. Louis, MO, USA, No.HPA023930), HSP90α (all at a 1:1000 dilution, Abcam); cyclin D1 (No. 55,506), CDK4 (No.12,790), CDK6 (No.13,331), Rb1 (No.9313), E2F1 (No.3742), GAPDH (No.5174) and HSP90α (No.4877) [all at a 1:1000 dilution purchased from Cell Signaling Technology, Boston, MA, USA].

### Immunoblot analysis

Cells were treated and harvested as described. The assay was performed as previously described [21]. Immunolabeling was visualized by an ECL (electrochemiluminescence) detection kit from Ammersham Biosciences according to the manufacturer’s instructions. The blots were from original gels which had to be cropped before hybridizing with secondary antibodies. GAPDH or HSP90α was used as a loading control.

### RNA interference and proliferation assays

Cell lines were transfected with short-interfering RNA (siRNAs, 30 nM final concentration) in 6-well plates with RNAiMAX (Invitrogen) according to the manufacturer’s instructions and harvested 48 hours after transfection, which could be cultured to enter following experiments. Target sequences for the siRNA of *ZNF703*: sense strand-5’ CCACACACUUUGGGCCUAA dTdT 3’; antisense-strand-3’ dTdT GGUGUGUGAAACCCGGAUU 5’. Non-targeting control siRNA was designed and synthesized by Guangzhou RuiBoBio (Guangzhou, China). Proliferation assay and colony-forming assay were performed as previously described [22]. Cell proliferation was measured by sulforhodamine B (SRB) (Sigma) assay. Relative growth was calculated as the value relative to controlled cells. In colony-forming assay, cells were seeded into 6-well plates (1000 cells per well). After several proper days, colonies were fixed in 10% acetic acid, 10% methanol and 80% ddH2O, and then stained with crystal violet (0.5% w/v).

### Cell cycle analysis

TNBC cells treated with non-targeting control siRNA or the siRNA of *ZNF703* were seeded in 6-well plates at a 60–70% confluence for 24 h. After that, TNBC cells were washed twice with PBS and fixed in 75% ethanol for 2 h at 4 ℃. Then, the TNBC cells were trypsinized and then suspended in fresh medium and centrifuged at 1,000 rpm for 5 min. Cell cycle analysis was performed as previously described [23]. The cells were washed with PBS and then stained with 0.05 µg/mL PI (Sigma-Aldrich), 1 µg/mL DNase-free RNase (Sigma-Aldrich) for 30 min. FACSCalibur analyzer (Becton-Dickinson, San Jose, CA, USA) was used to acquire events and Modfit software (Verity Software House, Topsham, ME, USA) was used to collect and analyze cell-cycle data.

### Immunohistochemistry Staining

Immunohistochemical analysis of tissue microarray sections were performed as previously described [22]. Tissue specimens were obtained from seventy-six patients who undergone surgical treatment at Ruijin Hospital (China) between January 2001 and December 2003 and were diagnosed of stage I-III primary breast cancer without history of other malignant tumors. Patients receiving chemotherapy or radiotherapy prior to surgery were excluded. Two pathologists were blinded to the clinicopathologic data and independently evaluated *ZNF703* expression as well as breast cancer subtype. As for *ZNF703*, they assessed the intensity of nuclear staining (0 score: no staining; 1 score: weak, 2 scores: moderate, 3 scores: strong) as well as the percentage of stained cells (0 score: 0%, 1 score: 1–20%, 2 scores: 21–40%, 3 scores: 41–60%, 4 scores: 61–80%, 5 scores: 81–100%). The final immunoreactive score ranged from 0 to 15, which equaled to the number of multiplying the intensity score by the percentage score. The median value was 5, by which it could divide patients into high expression group (above score 5), and low/no expression group (equal or below score 5). The study protocol was designed according to the principles of the Helsinki guidelines and approved by the institutional ethical board of Ruijin hospital affiliated to Shanghai Jiaotong university school of medicine. Cases were classified into two groups: low/no expression or high expression, according to median score of nucleic staining. The antibody was titrated with negative and positive controls. Evaluation of hormone receptor (HR) status accords with the Allred scoring method [24].

### Microarray data information from TCGA dataset and analysis

*ZNF703* mRNA expression data and corresponding clinical information of 136 basal-like invasive breast cancer samples, including basal-like 1 (BL1) and basal-like 2 (BL2) were obtained from The Cancer Genome Atlas (TCGA) dataset (https://portal.gdc.cancer.gov/) in January 2020, in which the method of acquisition and application complied with the guidelines and policies. Patients were divided into two groups according to the median value (Table 2), including *ZNF703*-low expression (seventy patients) and *ZNF703*-high expression (sixty-six patients) subgroups. Median follow-up was 9.5 years. The Kaplan-Meier survival analysis with log-rank test was used to compare the difference of overall survival between two groups [25, 26].

### Statistics

Data analysis was performed using the statistical package SPSS 26.0. Each experiment was repeated at least three times. Student’s t-test was used to evaluate numeric data. Chi-square test was used for comparisons of categorical data. For Kaplan–Meier curves, p-values, and hazard ratio (HR) with 95% confidence interval (CI) were generated by log-rank tests using GraphPad Prism (version 8.4.0). Statistical tests were two-sided, and *P-*values less than 0.05 were considered statistically significant.

## Results

### ZNF703 expression in TNBC

We detected the expression of *ZNF703* in thirteen breast cancer cell lines and two normal breast epithelial cell lines by western blot (Fig. 1A, Fig. S1). We found that normal breast epithelial cell line MCF-7-10 A did not express *ZNF703*. HBL-100 and most of the HER2-positve breast cancer cell lines such as BT-474, SK-BR-3 and ZR-7530 [27], expressed little *ZNF703* proteins. TNBC cell lines MDA-MB-435, MDA-MB-468, MDA-MB-231 and BT-549 expressed more amount of *ZNF703* proteins, although not at high levels. Luminal cell line MCF-7 and one HER2-positve cell line MDA-MB-453 also expressed a certain level of *ZNF703* proteins. We next selected BT-549 and MDA-MB-468 cell lines as the model to explore the role of *ZNF703 in vitro*. We also examined *ZNF703* expression in the tumor tissue block of 76 TNBC patients by immunohistochemistry (Fig. 1C, Table 1). Median age was 53 years old. Twenty-six cases (34.2%) with high expression of *ZNF703* were identified (Fig. 1B, C). *ZNF703* was not associated with age, grade, tumor size, lymph node metastases, stage and pathological type in those patients (*P* > 0.05). These findings mean that *ZNF703* expressed and could be detected in TNBC samples, whether in cell lines or in tumor specimen.

**Table 1:** IHC expression of *ZNF703* in seventy-six triple-negative breast cancer patients

Characteristics	Total No.	*ZNF703* Low/negative No. (%)	*ZNF703* High No. (%)	*P* value ^b^
**Age**	76			0.90
<=50		20 (40.0)	10 (38.5)	
>50		30 (60.0)	16 (61.5)	
**Lymph node metastases**	61 ^a^			0.14
No		31 (75.6)	11 (55.0)	
Yes		10 (24.4)	9 (45.0)	
**Grade**	76			0.46
1		2 (4.0)	3 (11.5)	
2		24 (48.0)	12 (46.2)	
3		24 (48.0)	11 (42.3)	
**Pathological type**	76			0.84
IDC		45 (90.0)	23 (88.5)	
other		5 (10.0)	3 (11.5)	
**Tumor size**	53 ^a^			
< 3 cm		15 (40.5)	8 (50.0)	0.56
>= 3 cm		22 (59.5)	8 (50.0)	
**Stage**	60 ^a^			
I		9 (22.5)	3 (15.0)	0.73
II/III		31 (77.5)	17 (85.0)	

^a^ Missing data not calculated statistically. ^b^ *P* values less than 0.05 considered statistically significant

### ZNF703 inhibition attenuates TNBC cell proliferation and colony formation

**Fig. 2:**
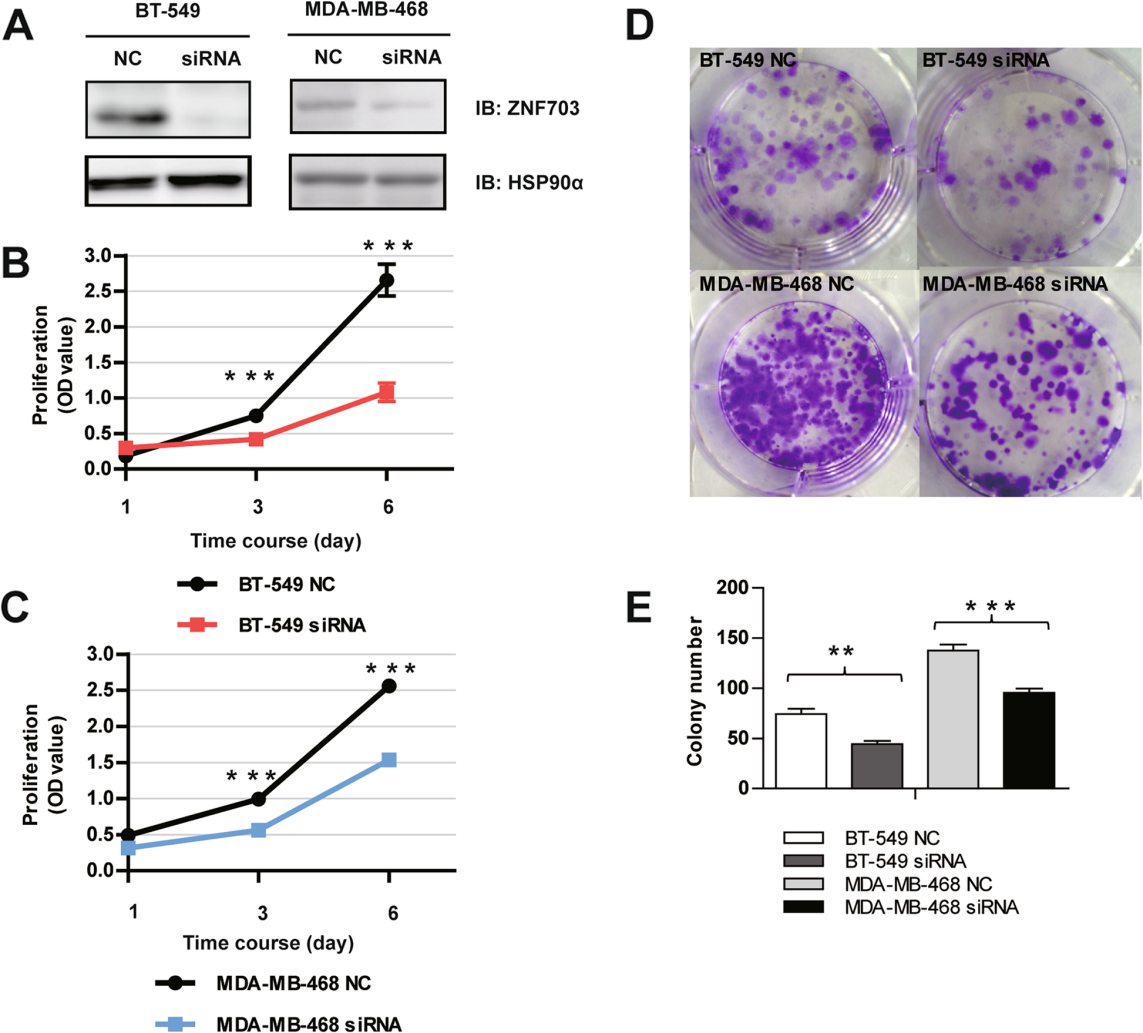
*ZNF703* knockdown affects the tumorigenesis of BT-549 and MDA-MB-468 cells. **A** Immunoblotting (IB) of *ZNF703* protein expression in BT-549 non-targeting siRNA control (NC), BT-549 siRNA, MDA-MB-468 NC, MDA-MB-468 siRNA cells. HSP90α was used as a loading control. **B** Growth curve of BT-549 NC and BT-549 siRNA cells. **C** Growth curve of MDA-MB-468 NC and MDA-MB-468 siRNA cells. Data are representative of three independent experiments and are presented as mean ± SD. **D**, **E** Cell growth was evaluated by the colony formation assay. Colony numbers were counted, and Fig. 2E represents an average of three independent experiments. (** *P* < 0.01, *** *P* < 0.001)

We established TNBC cell lines BT-549, MDA-MB-468 with non-targeting control siRNA (NC) or the siRNA of *ZNF703*, respectively (Fig. 2A). Next, we performed experiments to determine whether *ZNF703* could increase cell proliferation. The results showed that *ZNF703* inhibition could statistically significantly depress cell growth in a time-dependent model (Fig. 2B, C). We also performed a colony formation assay to verify the inhibitory effects of treatment with *ZNF703*-siRNA, as compared to control cells (Fig. 2D and E), with a statistically significant result.

### Anti-tumor effect of ZNF703 on TNBC through cell cycle signaling

**Fig. 3:**
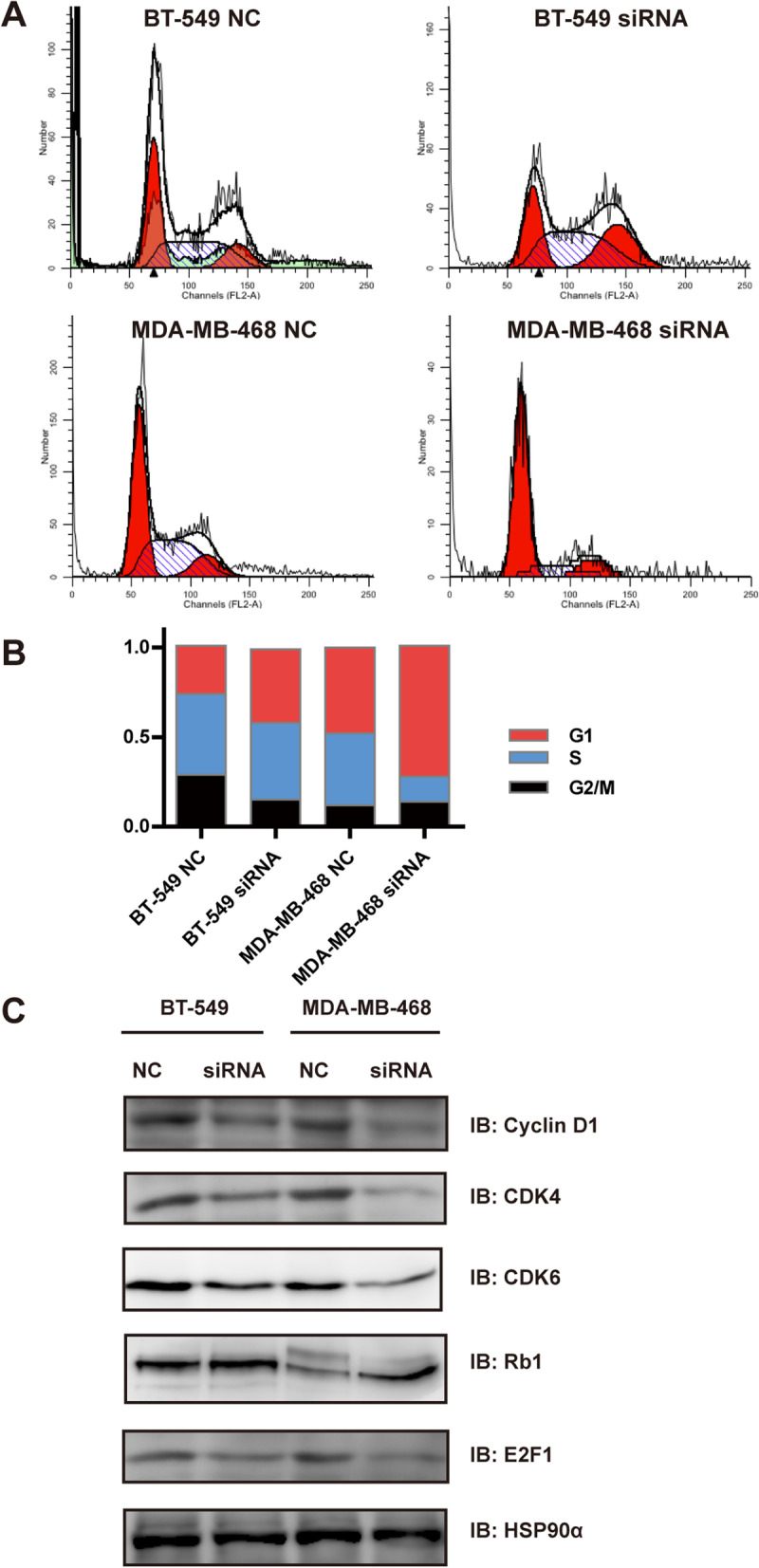
*ZNF703* regulates cell cycle of TNBC. **A**, **B** Inhibiting *ZNF703* induced G1-phase arrest in BT-549 and MDA-MB-468 cell lines. Cells were treated with NC or *ZNF703*-siRNA for 72 h, and DNA contents were detected and analyzed by flow cytometry assay. The percentage of cells in G1, S and G2/M of cell cycle were calculated. These results were from one representative experiment of three independent experiments. **C** Immunoblotting (IB) of lysates of BT-549 NC, BT-549 siRNA, MDA-MB-468 NC and MDA-MB-468 siRNA cells using the indicated antibodies. A HSP90α antibody was used as a loading control. The experiment was repeated for three times and one representative result was shown

To further evaluate the effect of *ZNF703* on cell growth, we tested the effect of *ZNF703*-siRNA on the cell cycle distribution of TNBC cells. As it was shown, in one representative experiment (Fig. 3A, B), the analysis revealed cell cycle distribution of NC-siRNA treated cells showing 26.75%, 47.97% in G1, 44.58%, 39.97% in S-phase, 28.67%, 12.06% cells in G2/M for BT549 and MDA-MB-468, respectively; while 41.47%, 72.59% in G1, 43.40%, 13.53% in S-phase, 15.13%, 13.88% cells in G2/M for BT549 and MDA-MB-468 cells treated with *ZNF703*-siRNA, respectively. The G1 phase fraction increased in BT-549 cells and MDA-MB-468 cells, after treating with *ZNF703*-siRNA, implying that in comparison with NC-siRNA treated cells, *ZNF703*-siRNA induced an accumulation of cells in the G1 phase fraction. Besides, after knockdown of *ZNF703*, we found that cyclin D1, CDK4 and CDK6, as well as E2F1, which played a role in the G1 phase of cell cycle regulation [28–30], were downregulated by immunoblotting, while the tumor suppressor gene Rb1 was upregulated (Fig. 3C, Fig. S2).

### Prognosis of ZNF703 expression in basal-like invasive breast cancer patients from TCGA

**Fig. 4:**
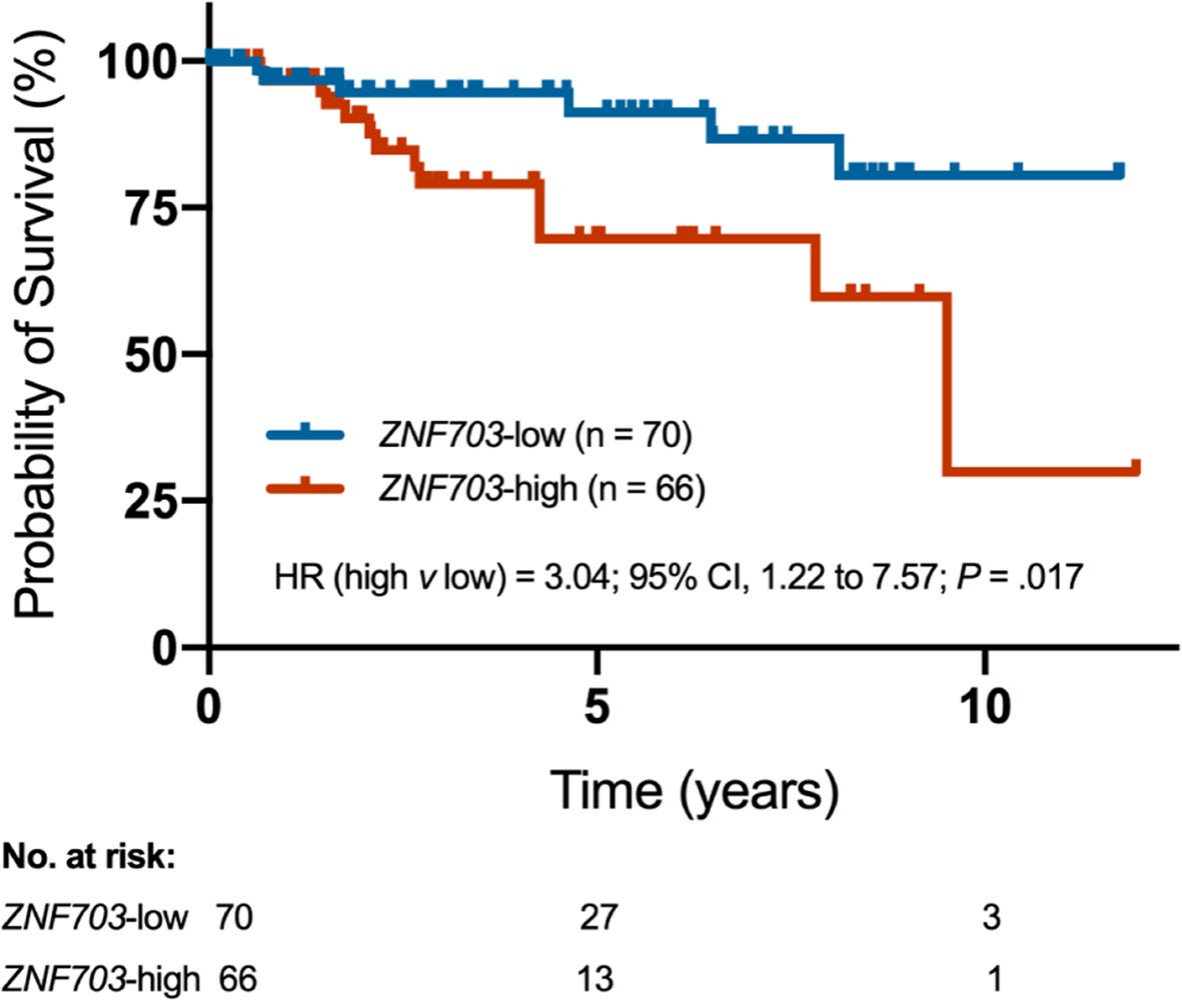
*ZNF703* mRNA expression predicts overall survival by Kaplan-Meier survival analysis. Median follow-up was 9.5 years. One hundred and thirty-six basal-like invasive breast cancer samples from TCGA were analyzed by Kaplan-Meier to compare the difference of overall survival between two groups. (HR, hazard ratio, 95%CI, 95% Confidence Interval)

TNBCs were classified into four transcriptomic subtypes, including basal-like 1 (BL1), basal-like 2 (BL2), mesenchymal (M) and luminal androgen receptor (LAR) [6, 31, 32]. Most of the TNBCs belong to basal-like subtypes. Here we collected and downloaded 136 basal-like invasive breast cancer samples from TCGA platform (Table 2). Median follow-up was 9.5 years. Kaplan-Meier survival analysis showed that (Fig. 4), high mRNA expression of *ZNF703* were statistically significantly correlated to worse overall survival (*HR* for high expression was 3.04; *95% CI*, 1.22 to 7.57, *P* = 0.017).

**Table 2:** *ZNF703* mRNA expression and clinicopathological characteristics in one hundred and thirty-six basal-like breast cancer patients from TCGA dataset

Sample ID	Age	pTNM_stage	Tumor Stage	Nodal Stage	*ZNF703* expression	Status	Time (years)
**TCGA-A1-A0SK-01**	<=60	II	T2	N0	High	Dead	2.65
**TCGA-A1-A0SO-01**	>60	II	T2	N1	Low	Alive	2.33
**TCGA-A1-A0SP-01**	<=60	II	T2	N0	High	Alive	1.60
**TCGA-A2-A04P-01**	<=60	III	T2	N3	High	Dead	1.50
**TCGA-A2-A04Q-01**	<=60	I	T1	N0	High	Alive	6.53
**TCGA-A2-A04T-01**	>60	II	T2	N0	High	Alive	6.15
**TCGA-A2-A04U-01**	<=60	II	T2	N0	Low	Alive	7.27
**TCGA-A2-A0CM-01**	<=60	II	T2	N0	High	Dead	2.07
**TCGA-A2-A0D0-01**	<=60	II	T2	N0	Low	Alive	5.61
**TCGA-A2-A0D2-01**	<=60	II	T2	N0	High	Alive	2.81
**TCGA-A2-A0ST-01**	>60	II	T1	N1	High	Alive	8.27
**TCGA-A2-A0SX-01**	<=60	I	T1	N0	High	Alive	4.20
**TCGA-A2-A0T0-01**	<=60	II	T2	N1	High	Alive	1.46
**TCGA-A2-A0T2-01**	>60	IV	T3	N3	Low	Dead	0.70
**TCGA-A2-A0YE-01**	<=60	II	T2	N1	High	Alive	1.52
**TCGA-A2-A0YJ-01**	<=60	III	T3	N2	Low	Alive	1.55
**TCGA-A2-A0YM-01**	>60	II	T2	N0	Low	Alive	2.64
**TCGA-A2-A1G1-01**	>60	II	T2	N1	High	Alive	1.60
**TCGA-A2-A25F-01**	>60	II	T2	N0	Low	Alive	0.88
**TCGA-A7-A0CE-01**	<=60	II	T2	N0	High	Alive	2.94
**TCGA-A7-A0DA-01**	>60	II	T2	N0	High	Alive	2.97
**TCGA-A7-A13D-01**	<=60	II	T2	N0	Low	Alive	2.64
**TCGA-A7-A13E-01**	>60	II	T2	N1	Low	Dead	1.68
**TCGA-A7-A26F-01**	<=60	I	T1	N0	Low	Alive	2.02
**TCGA-A7-A26G-01**	<=60	II	T2	N0	High	Alive	1.98
**TCGA-A7-A26I-01**	>60	II	T2	N0	Low	Alive	1.81
**TCGA-A8-A07C-01**	<=60	II	T2	N0	Low	Alive	2.83
**TCGA-A8-A07O-01**	<=60	II	T2	N0	Low	Alive	0.83
**TCGA-A8-A07R-01**	>60	III	T2	N3	Low	Alive	0.75
**TCGA-A8-A07U-01**	>60	III	T2	N2	Low	Alive	2.08
**TCGA-A8-A08R-01**	<=60	II	T2	N1	Low	Alive	0.08
**TCGA-AC-A2BK-01**	>60	III	T2	N2	High	Alive	6.09
**TCGA-AC-A2QH-01**	<=60	II	T3	N0	Low	Alive	2.75
**TCGA-AN-A04D-01**	<=60	II	T2	N1	Low	Alive	0.14
**TCGA-AN-A0AL-01**	<=60	III	T4	N0	High	Alive	0.62
**TCGA-AN-A0AT-01**	>60	II	T2	N0	Low	Alive	0.03
**TCGA-AN-A0FJ-01**	<=60	IV	T2	N2	High	Alive	0.66
**TCGA-AN-A0FL-01**	>60	II	T2	N0	High	Alive	0.63
**TCGA-AN-A0FX-01**	<=60	II	T2	N0	High	Alive	0.03
**TCGA-AN-A0G0-01**	<=60	II	T2	N0	High	Alive	0.04
**TCGA-AN-A0XU-01**	<=60	II	T2	N0	High	Alive	0.03
**TCGA-AO-A0J4-01**	<=60	I	T1	N0	Low	Alive	4.35
**TCGA-AO-A0J6-01**	>60	II	T2	N0	Low	Alive	3.12
**TCGA-AO-A0JL-01**	<=60	III	T2	N2	Low	Alive	4.61
**TCGA-AO-A124-01**	<=60	II	T2	N0	Low	Alive	9.61
**TCGA-AO-A128-01**	>60	II	T2	N0	Low	Alive	8.90
**TCGA-AO-A129-01**	<=60	II	T2	N1	Low	Alive	9.00
**TCGA-AO-A12F-01**	<=60	II	T2	N0	High	Alive	5.05
**TCGA-AO-A1KR-01**	<=60	II	T2	N0	Low	Alive	6.88
**TCGA-AQ-A04J-01**	<=60	II	T2	N0	High	Alive	2.24
**TCGA-AR-A0TP-01**	<=60	II	T2	N0	Low	Alive	11.71
**TCGA-AR-A0TS-01**	<=60	II	T2	N1	Low	Alive	7.09
**TCGA-AR-A0TU-01**	<=60	II	T2	N0	High	Alive	1.94
**TCGA-AR-A0U0-01**	>60	II	T2	N1	Low	Alive	5.45
**TCGA-AR-A0U4-01**	<=60	II	T2	N0	Low	Alive	8.93
**TCGA-AR-A1AH-01**	<=60	II	T2	N1	Low	Alive	10.43
**TCGA-AR-A1AI-01**	<=60	II	T2	N0	Low	Alive	9.03
**TCGA-AR-A1AJ-01**	>60	I	T1	N0	Low	Alive	8.42
**TCGA-AR-A1AQ-01**	<=60	II	T2	N0	High	Alive	8.28
**TCGA-AR-A1AR-01**	<=60	III	T1	N2	High	Dead	1.44
**TCGA-AR-A1AY-01**	>60	I	T1	N0	High	Alive	2.81
**TCGA-AR-A24Q-01**	<=60	II	T3	N0	Low	Alive	8.69
**TCGA-AR-A251-01**	<=60	III	T2	N2	Low	Alive	8.30
**TCGA-AR-A256-01**	<=60	II	T2	N0	High	Dead	7.82
**TCGA-AR-A2LR-01**	<=60	I	T1	N0	High	Alive	4.77
**TCGA-B6-A0I1-01**	>60	II	T2	N0	Low	Dead	6.47
**TCGA-B6-A0I2-01**	<=60	I	T1	N0	High	Alive	11.95
**TCGA-B6-A0I6-01**	<=60	II	T1	N1	High	Dead	2.72
**TCGA-B6-A0IQ-01**	<=60	III	T3	N1	Low	Alive	11.74
**TCGA-B6-A0RT-01**	<=60	III	T3	N1	Low	Alive	7.45
**TCGA-B6-A0WX-01**	<=60	III	T3	N1	High	Dead	1.75
**TCGA-B6-A1KF-01**	>60	II	T2	N1	High	Alive	8.46
**TCGA-BH-A0AV-01**	<=60	I	T1	N0	High	Alive	4.99
**TCGA-BH-A0B3-01**	<=60	II	T2	N1	High	Alive	3.30
**TCGA-BH-A0BG-01**	>60	I	T1	N0	Low	Alive	5.13
**TCGA-BH-A0BL-01**	<=60	I	T1	N0	High	Alive	6.24
**TCGA-BH-A0BW-01**	>60	I	T1	N0	Low	Alive	6.50
**TCGA-BH-A0DL-01**	>60	II	T2	N0	High	Alive	6.52
**TCGA-BH-A0E0-01**	<=60	III	T3	N3	Low	Alive	0.37
**TCGA-BH-A0E6-01**	>60	I	T1	N0	Low	Alive	0.80
**TCGA-BH-A0RX-01**	<=60	II	T2	N0	High	Alive	0.47
**TCGA-BH-A0WA-01**	>60	I	T1	N0	High	Alive	1.92
**TCGA-BH-A18G-01**	>60	I	T1	N0	Low	Alive	0.41
**TCGA-BH-A18Q-01**	<=60	II	T2	N1	Low	Dead	4.64
**TCGA-BH-A18T-01**	>60	II	T2	N0	Low	Dead	0.61
**TCGA-BH-A18V-01**	<=60	II	T2	N1	High	Dead	4.26
**TCGA-BH-A18V-06**	<=60	II	T2	N1	High	Dead	4.26
**TCGA-BH-A1F0-01**	>60	II	T1	N1	High	Dead	2.15
**TCGA-BH-A1F6-01**	<=60	NA^a^	T4	N2	Low	Dead	8.12
**TCGA-BH-A1FC-01**	>60	II	T1	N1	High	Dead	9.51
**TCGA-C8-A12K-01**	>60	II	T2	N1	Low	Alive	0.01
**TCGA-C8-A12V-01**	<=60	II	T2	N0	High	Alive	1.05
**TCGA-C8-A131-01**	>60	III	T2	N2	High	Alive	1.12
**TCGA-C8-A134-01**	<=60	II	T2	N0	High	Alive	1.05
**TCGA-C8-A1HJ-01**	<=60	II	T2	N0	Low	Alive	0.01
**TCGA-C8-A27B-01**	<=60	II	T3	N0	Low	Alive	1.20
**TCGA-D8-A142-01**	>60	II	T3	N0	High	Alive	1.16
**TCGA-D8-A143-01**	<=60	II	T2	N0	High	Alive	1.18
**TCGA-D8-A147-01**	<=60	II	T2	N0	Low	Alive	1.60
**TCGA-D8-A1JK-01**	>60	II	T2	N0	High	Alive	1.68
**TCGA-D8-A1JL-01**	>60	II	T2	N0	High	Alive	1.67
**TCGA-D8-A1JM-01**	<=60	II	T2	N1	Low	Alive	1.61
**TCGA-D8-A1XK-01**	<=60	II	T2	N1	Low	Alive	1.2
**TCGA-D8-A1XQ-01**	>60	II	T2	N0	High	Alive	1.37
**TCGA-D8-A27F-01**	<=60	II	T2	N0	High	Alive	1.34
**TCGA-D8-A27H-01**	>60	II	T2	N0	High	Alive	1.09
**TCGA-D8-A27M-01**	<=60	I	T1	N0	Low	Alive	1.12
**TCGA-E2-A14N-01**	<=60	II	T2	N1	Low	Alive	3.93
**TCGA-E2-A14R-01**	>60	II	T2	N0	Low	Alive	3.22
**TCGA-E2-A14X-01**	<=60	III	T2	N2	Low	Alive	2.66
**TCGA-E2-A14Y-01**	<=60	II	T2	N0	Low	Alive	5.78
**TCGA-E2-A150-01**	<=60	II	T2	N0	Low	Alive	5.30
**TCGA-E2-A158-01**	<=60	II	T1	N1	Low	Alive	1.23
**TCGA-E2-A159-01**	<=60	II	T2	N0	High	Alive	2.09
**TCGA-E2-A1AZ-01**	>60	II	T2	N1	Low	Alive	6.38
**TCGA-E2-A1II-01**	<=60	I	T1	N0	High	Alive	2.81
**TCGA-E2-A1LG-01**	<=60	II	T2	N0	High	Alive	4.17
**TCGA-E2-A1LH-01**	<=60	I	T1	N0	Low	Alive	8.90
**TCGA-E2-A1LI-01**	<=60	II	T2	N1	Low	Alive	8.55
**TCGA-E2-A1LK-01**	>60	III	T4	N3	High	Dead	0.73
**TCGA-E2-A1LL-01**	>60	III	T3	N2	High	Alive	3.59
**TCGA-E2-A1LS-01**	<=60	I	T1	N0	Low	Alive	4.39
**TCGA-E9-A1N8-01**	<=60	II	T2	N0	Low	Alive	2.85
**TCGA-E9-A1N9-01**	<=60	II	T2	N0	High	Alive	3.02
**TCGA-E9-A1ND-01**	>60	II	T2	N1	Low	Alive	3.47
**TCGA-E9-A22G-01**	<=60	II	T2	N0	Low	Alive	3.39
**TCGA-E9-A243-01**	<=60	II	T2	N0	Low	Alive	1.68
**TCGA-E9-A244-01**	<=60	II	T2	N0	Low	Alive	0.06
**TCGA-EW-A1OW-01**	<=60	II	T2	N0	High	Alive	1.90
**TCGA-EW-A1P4-01**	<=60	II	T2	N0	High	Alive	2.48
**TCGA-EW-A1P8-01**	<=60	III	T2	N3	High	Dead	0.65
**TCGA-EW-A1PB-01**	>60	III	T3	N1	High	Alive	1.67
**TCGA-EW-A1PH-01**	<=60	II	T1	N1	High	Alive	1.66
**TCGA-GI-A2C9-01**	<=60	II	T3	N0	High	Alive	9.16
**TCGA-GM-A2DF-01**	<=60	II	T1	N1	Low	Alive	5.9
**TCGA-HN-A2NL-01**	<=60	II	T2	N0	Low	Alive	0.22

^a^ *NA* not available

## Discussion

TNBC accounts for 15–20% of newly diagnosed breast cancer cases [1] and lacks effective treatment options. The combination of a biomarker-based paradigm and a subtyping-based paradigm is recommended to prompt a suitable targeted treatment for individual TNBC [33]. Although next-generation sequencing technology has facilitated identifying potential targets, few of the findings have been translated into daily clinical practice for treating TNBC patients.

In a formalin-fixed paraffin-embedded (FFPE)-based next-generation sequencing (NGS) analysis in the Neoadjuvant GeparSepto Trial [34], high genetic heterogeneity was observed in different breast cancer types. In this most recent study, *ZNF703* amplification occurred in 18.2% of triple-negative breast cancer patients, indicating the potential role played in TNBC development. In another study, which explored the associations between gene mutations and clinicopathologic characteristics by FoundationOne CDx assay in a cohort of 223 clinically advanced breast cancers, *ZNF703* gene alterations were enriched in 7.2% of locally advanced TNBCs, but not in metaplastic TNBCs [35]. However, the inner mechanisms have not been investigated in these studies. In our study, 34.2% of TNBC patients with high expression of *ZNF703* were identified by immunohistochemistry. There is a low correlation between amplification and overexpression in amplicon genes, and the amplicon does not influence tumor mutation burden in breast cancers [20]. Thus, intermediate, or even low expressions of genes are still likely to have an effect on tumor biology behaviors.

Besides luminal B breast tumors, *ZNF703* was also reported to have been implicated in infiltrating lobular breast cancer or progression of lobular carcinoma in situ to invasive cancer [36]. One study showed that *ZNF703* was a target of long noncoding RNA SPRY4-IT1 and played an oncogenic role in ER-negative breast cancer cells [37]. Furthermore, *ZNF703* seemed to be associated with PR loss, exhibiting more *ZNF703* amplification events in ER+PR-HER2- breast tumors than ER+PR+HER2- breast tumors [38]. These studies indicate that *ZNF703* can influence the tumorigenesis of different kinds of breast cancer types, not only on the luminal B breast cancer. Levisticum officinale, an herbal plant, was proved to have anti-proliferative and apoptotic activities in a TNBC cell line, with higher expression of *ZNF703* than in the less invasive MCF-7 cells [39]. In our study, we demonstrated that *ZNF703* inhibition suppressed cell proliferation and cell cycle in two TNBC cell lines, indicating its expression regulates these processes. It is interesting that G1-phase arrest could be induced by inhibiting *ZNF703* in TNBC cell lines, which is the new mechanism that was observed for *ZNF703* in the context of TNBC. *ZNF703* could have influences on several vital cell-cycle related proteins or kinases, such as cyclin D1, CDK4, CDK6 and Rb1, which triggered the changes of most important downstream transcriptional factor E2F1. However, there is a limitation that this result may need to be verified *in vivo* experiments in the future. In addition, inner mechanisms of how *ZNF703* functions in cell cycle, for instance, through epigenetic molecules or protein-protein interactions, and the application of cell-cycle inhibitors like CDK4/6 inhibitor in combination with *ZNF703* inhibitor, could be further explored.

## Conclusions

Collectively, for the first time, our findings revealed that *ZNF703* was a potentially vital protein for TNBC. Targeting *ZNF703* contributed to the anti-tumor effects in TNBC cells through G1-phase arrest. *ZNF703* can be explored as a novel therapeutic target for TNBC in further clinical trials.

## Supplementary Information

**Additional file 1: Figure S1.** Quantification of the western blot analysis in Fig. 1A. ZNF703 expressions of different breast cell lines in Fig. 1A were measured using ImageJ software and normalized to GAPDH levels.

**Additional file 2: Figure S2.** Quantification of the western blot analysis in Fig. 3C. Expressions of Cyclin D1, CDK4, CDK6, Rb1 and E2F1 in Fig. 3C were measured using ImageJ software and normalized to HSP90α levels.

## Data Availability

The data that support the findings of this study are available from The Cancer Genome Atlas (TCGA) dataset (https://portal.gdc.cancer.gov/) and the corresponding author [X. Z] upon reasonable request.

## Abbreviations

ZNF703: Zinc finger 703
TNBC: Triple negative breast cancer
ERα: Estrogen receptorα
PR: Progesterone receptor
HER2/ERBB2: Human epidermal growth receptor 2/ erb-b2 receptor tyrosine kinase 2
siRNA: Short interfering RNA
TCGA: The Cancer Genome Atlas
CDK4: Cyclin-dependent kinase 4
CDK6: Cyclin-dependent kinase 6
E2F1: E2F transcription factor 1
Rb1: Human retinoblastoma susceptibility gene
