# Supplementary figures and images for: ZNF703 promotes triple-negative breast cancer cells through cell-cycle signaling and associated with poor prognosis
Source: BMC Cancer. 2022 Mar 2;22:226. doi: 10.1186/s12885-022-09286-w (PMC8889678; doi:10.1186/s12885-022-09286-w)

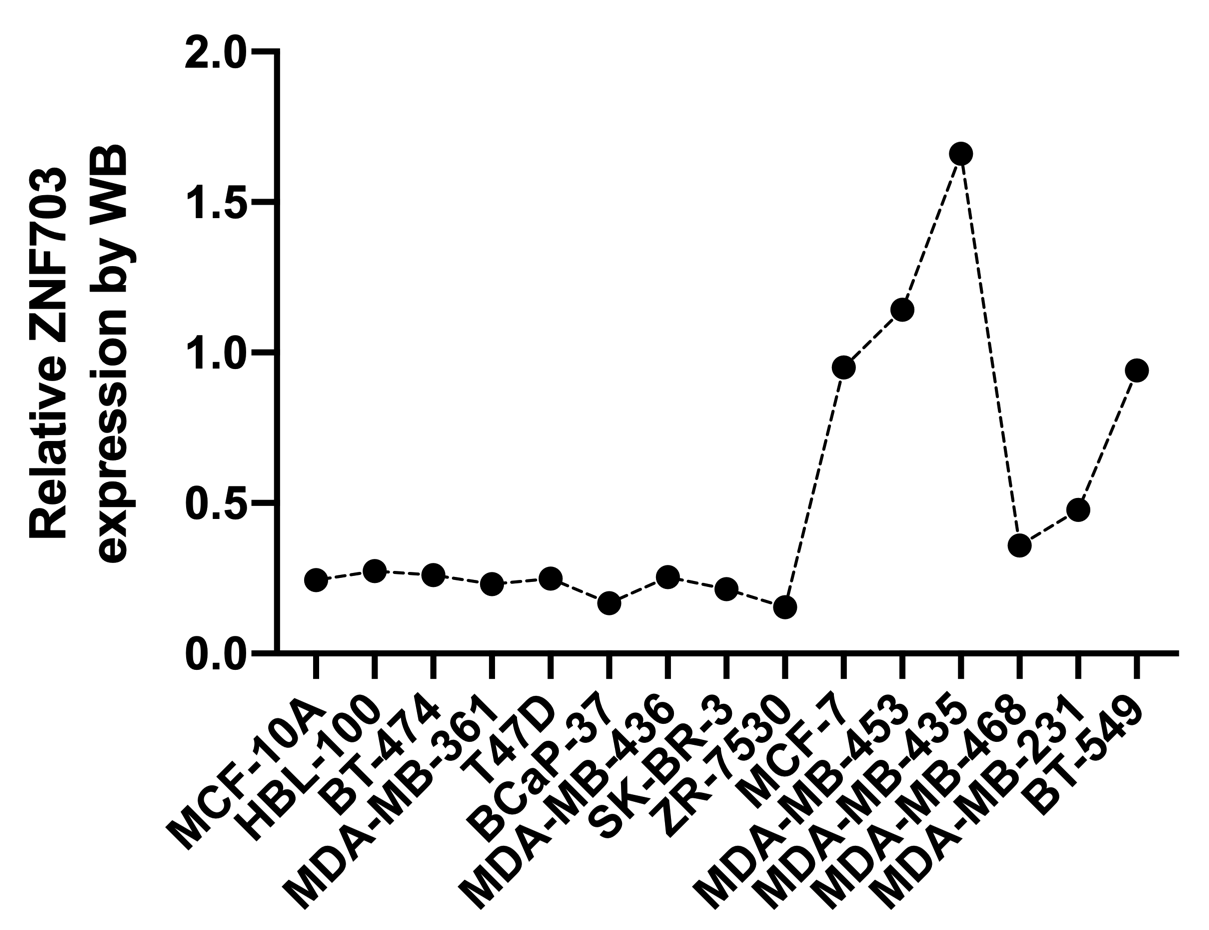

Supplement: Supplementary file 1 — Additional file 1: Figure S1. Quantification of the western blot analysis in Fig. 1A. ZNF703 expressions of different breast cell lines in Fig. 1A were measured using ImageJ software and normalized to GAPDH levels. [file 12885_2022_9286_MOESM1_ESM.tiff]

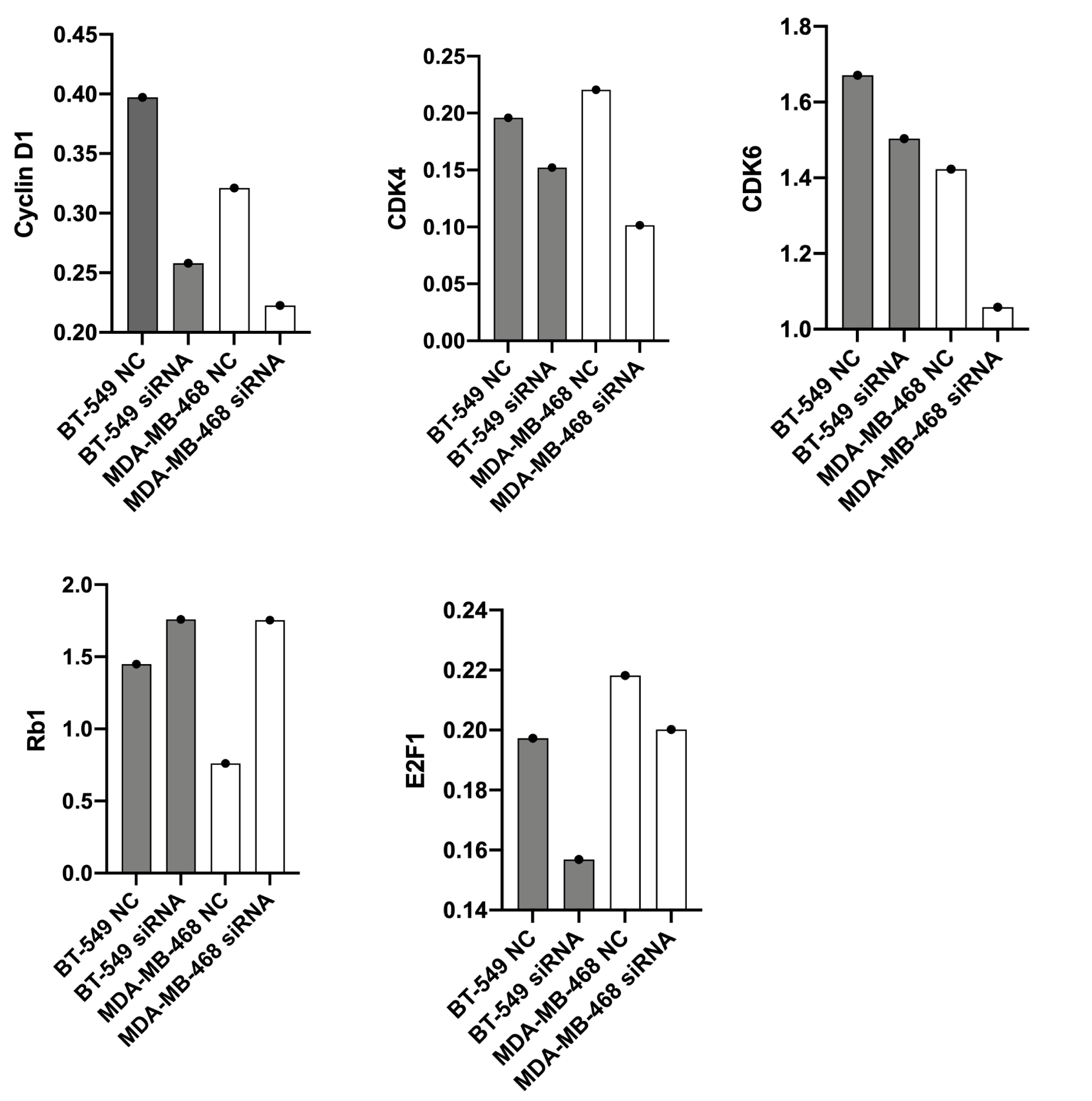

Supplement: Supplementary file 2 — Additional file 2: Figure S2. Quantification of the western blot analysis in Fig. 3C. Expressions of Cyclin D1, CDK4, CDK6, Rb1 and E2F1 in Fig. 3C were measured using ImageJ software and normalized to HSP90α levels. [file 12885_2022_9286_MOESM2_ESM.tif]
